# Low-Cost Electronic Microwave Calibration for Rapid On-Line Moisture Sensing of Seedcotton

**DOI:** 10.3390/s101211088

**Published:** 2010-12-06

**Authors:** Mathew G. Pelletier, Joseph A. Viera

**Affiliations:** 1 United States Department of Agriculture; Agricultural Research Services, Lubbock, TX 79403, USA; 2 Sensors Group Microsemi Corporation Lowell, MA 01851, USA; E-Mail: jviera@microsemi.com

**Keywords:** cotton, moisture, sensing, microwave

## Abstract

In order to improve rapid on-line moisture sensing of seedcotton in cotton gins, a means by which to establish a reliable low-cost wide-band electronic calibration is critically needed. This calibration is needed to center the circuit due to changes in the internal signal delays and attenuation drift caused by temperature changes in the various system components and circuit elements. This research examines a hardware technique for use in conjunction with microwave reflective sensing probes having an extended bandwidth from 500 MHz through 2.5 GHz. This new technique was validated experimentally against known electrical propagation delay standards. Results of the measured propagation delay with this type of automatic electronic calibration method was found to agree with results using a vector network analyzer with a traditional S11 single port error correction calibration methodology to within 4% of the measurement, 95% confidence, with a standard error of +/− 18.6 ps for the delay measurements. At this level of performance, the proposed low-cost technique exhibits superior performance, over the typical geosciences time-domain reflectometer “TDR”, instruments in common use in soil moisture testing and is suitable for use in cotton gin moisture sensing.

## 1. Introduction

As the demand for energy escalates due to the build up of the third world economies, the cost of energy is exerting an escalating pressure on the ability of cotton processors to provide a high quality product at a competitive cost. Improved energy efficiency is critically needed as a way to reduce processing costs. Cotton drying is a major component of the overall cotton production process that has one of the highest energy demands, so there is a critical need for development of optimal drying systems to maximize the energy efficiency of the drying process. This need in turn dictates a need for high quality, high speed on-line sensors for accurate determination of moisture content, as needed to optimally control the driers.

**Figure 1.**
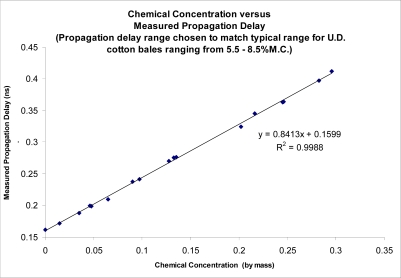
Sensor’s performance tested against known dielectric permittivity standards to determine accuracy of antennas for free space permittivity sensing.

**Figure 2.**
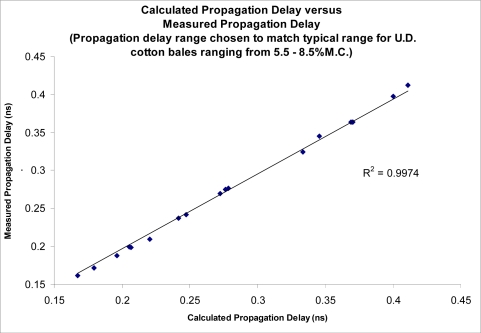
Comparison between permittivity as calculated from the known values versus sensor’s measured permittivity.

In commercial cotton gin processing, seedcotton is brought into the gins at various stages of moisture. Currently the seedcotton sensors available to sense the moisture content are limited to a few very low frequency, direct current (DC) resistance based sensors. Unfortunately, these sensors are subject to large errors caused by a wide variety of sources such as Maxwell-Wagner polarization and presence of surface salts and metal ions in dust dissolved in surface moisture. The use of a sensing frequency in the microwave region is known to minimize the impact of most of these errors with the added advantage of providing a strong correlation between the propagation delay of the microwave signal, and the moisture-density product of the lint cotton. This sensing technique has been tested using solutions of known permittivity, as well as on mini-cotton bales. The sensing technique demonstrated the ability to accurately determine permittivity of materials, as reported by Pelletier, [1–5]. Results are reproduced here for the convenience of the reader (Figures 1 and 2).

The primary goal of this research was to develop optimal drier performance, by seeking to develop a robust sensor that will provide the ability to more accurately sense seedcotton moisture content. The primary objective of this phase of the research was to examine ways to transfer the previous microwave cotton bale moisture sensor techniques for use in on-line sensing of seedcotton moisture, in order to provide more nearly optimal control of the drying systems in cotton gins.

## 2. Background

**Figure 3.**
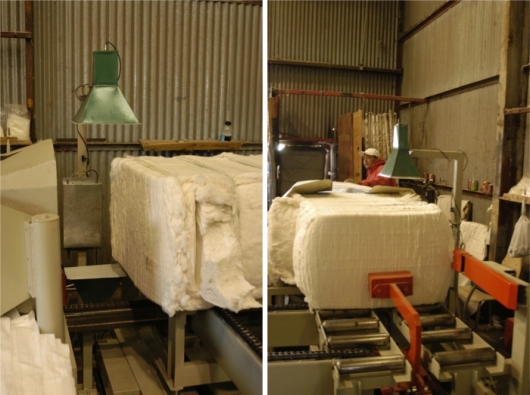
Shows how a bale is typically conveyed past the sensing station of one of the author’s original prototype microwave moisture sensing systems.

This research built on the earlier success of this laboratory’s microwave moisture sensors [1–5] to develop a method for imaging internal cotton bale moisture (Figure 3).

In the current configuration of the moisture sensors, for imaging internal cotton bale moisture, once the bale has cleared the sensing antennas, the system takes an air-reference reading by which to remove drift and bias from the circuit. This is necessary because of changes in the internal signal delays and attenuations caused by temperature changes to the various system components and circuit elements. This technique has been very successful for imaging internal cotton bale moisture, but unfortunately, for many optimal locations in cotton gins that need to be used to sense seedcotton moisture, there is no way to physically remove the seedcotton material from the on-line system to allow for periodic calibration of the circuit. Further, due to the extremely wide seasonal/diurnal temperature fluctuations in a cotton gin, the lack of availability of a calibration reference imposes severe errors onto the sensing system, thereby causing significant deterioration of the desired moisture sensing accuracy. To alleviate this problem, this research examined a hardware technique for electronic internal calibration for use in conjunction with microwave reflective sensing probes having an extended bandwidth from 500 MHz through 2.5 GHz.

## 3. Theory

In a free space measurement, the propagation of a free-space electromagnetic plane wave can be modeled by solving Maxwell’s electromagnetic equations (Equations 1 and 2), for plane wave propagation in a source-less region that is directed only in the z direction.

(1) $$\frac{\partial \text{H}}{\partial t}=-\frac{1}{\mu }\nabla \times \text{E}$$

(2) $$\frac{\partial \text{E}}{\partial t}=\frac{1}{ɛ}\nabla \times \text{H}-\frac{\sigma }{ɛ}\text{E}$$

where

∇ : Gradient Operator
E: Electric Field (V/m)
H: Magnetic Field (A/m)
*ɛ*: Permittivity of medium
*μ*: permeability of medium
σ: conductivity of medium

and boldface type is used to indicate vectors. The solution of these equations shows the plane wave propagation to have the form of Equation 3 [6],

(3) $${\text{e}}^{\gamma }=e^{jk}=e^{\alpha +j\beta }$$

with the propagation coefficient γ as shown in Equation 4 [6].

(4) $$\gamma =jk=\alpha +j\beta =j\sqrt{\varpi^{2}\mu ({ɛ}^{\prime }-j\frac{\sigma }{\varpi }-j{ɛ}^{\prime \prime })}=j\sqrt{\varpi^{2}\mu {ɛ}^{\prime }(1-j\;\text{tan}\delta )}$$

**Figure 4.**
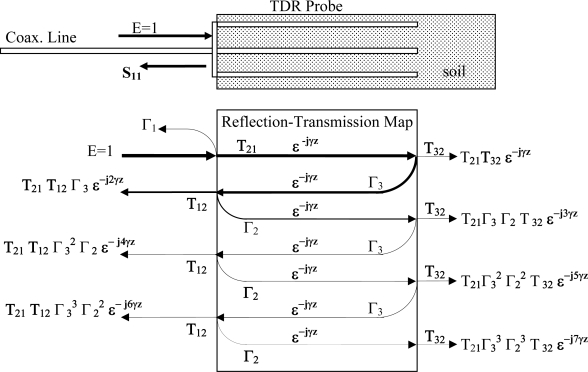
Detail of resultant waveform from combination of multiple reflections from both the leading edge (undesired) and probe end (desired) measurement, in TDR/FDR probes due to impedance mismatch between coaxial cable impedance Z_o_ to the soil-probe impedance Z_1_. Note: Hatched area indicates soil or other material under test.

However, when the system is translated from a free space measurement to a reflectance probe measurement, the reflectance probe introduces an impedance mismatch onto the measurement. This mismatch varies with the value of the complex permittivity of the material that occupies the space inside the reflectance probe. This impedance mismatch causes multiple reflections to be set up in the measurement system as shown in Figure 4.

These multiple reflections, depending upon the magnitude of the mismatch, have the potential to lead to large errors that are dependent upon the frequency as well as the material’s complex permittivity, since that permittivity defines, along with the geometry of the sensing structure, the impedance of the sensing structure. A similar situation is created if another circuit, for use in automatic calibration, is inserted between the sensing reflection probe and the measurement circuitry.

As noted above, Equations 1 and 2 are sufficient to provide a means to measure complex permittivity for free-space measurements, however, when measurements are taken with a TDR or similar reflection probe, the impedance mismatch modifies how the plane wave propagates inside the waveguide and how it is subsequently returned for observation and determination of moisture. Thus, for accurate results, in order to obtain a measurement of the true absolute permittivity, rather than a measure of the apparent permittivity provided by a simple measurement of the attenuation and phase delay, it is necessary to also characterize the waveguide’s frequency response effect on the plane-wave, in order to extract the real permittivity from a measurement taken with a coaxial probe. We note here that the act of insertion of yet another structure for automatic calibration will modify this frequency response and may need to be accounted for in the final model.

To find the response when the medium is inside a coaxial cable, the formulation must be converted from electric and magnetic fields to voltage, current and impedance. The impedance of a coaxial cable can be shown to be [7]:

(5a) $$Z_{c}=\frac{\eta }{2\pi }\text{ln}(\frac{b}{a})$$

(5b) $$\eta =\sqrt{\frac{\mu_{0}}{ɛ}}$$

where

Z_C_ = impedance of the TDR probe with non-permeable medium and complex permittivity ɛ.
*η* = impedance of dielectric medium filling coaxial core between inner and outer conductors.
b = outer diameter of coaxial core
a = inner diameter of coaxial core

Next we note that for a given geometry, Z_C_ will not match Z_0_ (impedance of the measurement system and inter-connecting cable). Due to this mismatch between Z_C_ and Z_0_, a partial reflection of the incoming wave will take place at the front edge between the coaxial cable connector at the beginning of the measurement zone. Thus, at the interface between the cable and coaxial media-under-test waveguide, there will be a reflection back towards the source. Further, the partially transmitted wave will then proceed to the end of the coaxial cell where it will reflect back towards the front edge, where the impedance mismatch will again cause a partial reflection such that the wave has to propagate through the cell a 2nd time and so on. This leads to multiple internal reflections, as shown in Figure 4.

The magnitude of the first reflection at the interface can be shown to be given by Equations 6(a,b) [6].

(6a) $$\Gamma_{1}=\frac{Z_{1}-Z_{0}}{Z_{1}+Z_{0}}$$

(6b) $$\Gamma_{2}=\frac{Z_{0}-Z_{1}}{Z_{1}+Z_{0}}$$

where

Γ_1_ = reflection coefficient at transition from cable to TDR probe, Figure 1.
Γ_2_ = reflection coefficient at transition from TDR probe to cable, Figure 1.
Z_0_ = impedance of the coaxial cable connecting soil probe to instrumentation.
Z_1_ = impedance of the coaxial soil probe, Equation 15a.

We also note from figure 4, that in addition to the first reflection back towards the source, an additional reflection will take place, such that the multiple reflection combinations provide the frequency response reflection coefficient that the TDR is actually measuring, as given in Equation 7.

(7) $$\Gamma_{\text{measured}}=\Gamma_{1}+T_{2}T_{1}\Gamma_{3}e^{-2j\gamma z}+T_{2}T_{1}\Gamma_{2}\Gamma_{3}^{2}e^{-4j\gamma z}+T_{2}T_{1}\Gamma_{2}^{2}\Gamma_{3}^{3}e^{-6j\gamma z}+\ldots$$

γ = propagation constant for the medium, Equation 1.
z = propagation distance through the medium (m)
Γ_1_ = reflection coefficient off transition from cable to TDR probe, Figure 1.
Γ_2_ = reflection coefficient off transition from TDR probe to cable, Figure 1.
Γ_3_ = reflection coefficient off far tip of TDR probe, Figure 1.
T_1_ = transmission coefficient from transition from cable to TDR probe, Figure 1.
T_2_ = transmission coefficient from transition from TDR probe to cable, Figure 1.

Simplifying provides the final closed form solution relating the measured reflection coefficient Γ_measured_ to the desired free space propagation constant γ that is required for determination of the true material permittivity) by noting Equation 7 can be expressed as an infinite sum leads to the solution shown in Equation 8, [6].

(8) $$\Gamma_{\text{measured}}=\frac{\Gamma_{1}+\Gamma_{3}e^{-2j\gamma z}}{1+\Gamma_{1}\Gamma_{3}e^{-2jyz}}$$

In summary, it can be seen from Equations 1–8, which as the media under test surrounding the reflectance probe becomes wetter or drier, the impedance mismatch changes and must be corrected through the use of Equation 8 to arrive at the correct measure of permittivity from a given measure of apparent permittivity. We note herein that our objective to utilize a low cost electronic-calibration circuit is compromised by the use of an imperfectly matched test cell, thus the primary objective of this research is to quantify the loss of accuracy of a low cost high-bandwidth CMOS single-pole to 4-throw switch by which to select from either the sensing probe with a material of unknown permittivity, or one of the calibration standards such as an open, short or 50-Ohm load, per traditional network analyzer style calibration protocols. In order to isolate the impact of associated errors to only the impact created by the use of a semi-conductor switch used to select into the measurement circuit one of the calibration standards, the criteria for selection of precision delay lines for quantifying the performance were chosen;

- as they are readily characterized by the Network Analyzer for a high accuracy estimate of the true delay,
- as they come with manufacturer specifications stating the expected delays for each line,
- for the materials of interest, the impedance miss-match creates errors that are potentially as large or larger than the errors that a good low cost electronic calibration circuit would create; thus, the use of actual media does not provide the best methodology for evaluation of a low cost calibration system.

Noting that the primary goal of this research is a low cost electronic calibration circuit, thus of interest are the various types of network calibration techniques. However, given the wealth of information readily available in the literature, the reader is directed to the following references [8–12], rather the discussing in depth here. Furthermore, while the authors acknowledge the abundance of literature directed towards calibration techniques, all of the papers the authors discovered were directed towards high quality calibration systems suitable for high-end vector network analyzers, not towards low cost sensors designed for process control.

Also, of principal note to this work, is the expected use of reflectance cells, thus of interest is to find a low cost calibration system suitable for single port calibrations such as one-port short, open, load, (SOL) calibrations that are used to establish the 3-term error corrections required to remove the internal systematic and drift errors from the one-port reflection measurements for frequency domain sensors.

## 4. Methods

The core of the proposed auto-calibration circuit is for the potential use of a readily available off the shelf microwave switch to automatically switch in and out the various calibration standards (open, short and a load). The microwave switch selected for this test was based upon CMOS process that yielded a wide 2.5 GHz bandwidth switch that exhibited greater than 37 dB of isolation between circuits at 1 GHz. In envisioned use, the proposed calibration circuit would be inserted between the sensing system and the material sensing reflectance probe, immediately before the reflectance probe.

To test the validity, and impact imposed by the inserted calibration circuit; a test was designed to compare the measured time as the signal traverses the precision delay lines of varying lengths, thereby simulating the delay incurred when the permittivity of a low-loss material increases which also increases the signal delay. The range of delay times, that the delay lines imposed, were chosen to simulate a wide range of permittivity’s that are felt to be representative of the expected delays that are anticipated for use in moisture sensing for the materials of interest in the targeted reflection cell. The initial control, by which to judge quality of the proposed calibration circuit, were measurements that were obtained from a direct measurement using a microwave vector network analyzer, using a calibration protocol that set the calibration reference plane at the connector entrance to the precision delay lines. The calibration was performed via a 1-port S11 standard correction technique utilizing (a) open, (b) short and (c) a load. As the delay lines were also connected at the face of the reference plane, the calibration effectively allows for measurement of the delay that is limited to only the delay created by the precision delay lines. After obtaining the calibration, a set of 6 delay lines were tested: 1,074 ps, 1,543 ps, 1,795 ps, 2,022 ps, 2,162 ps, 3,226 ps. As mentioned previously, these delay times were selected to be representative of the expected delay range of a typical installation for our target sensor. Each delay line was measured for S11 parameters at the following frequencies; {500, 1,000, 1,500, 2,000, 2,500 MHz}. The phase delay was subsequently unwrapped to provide an unambiguous phase delay. Each of these gold standard network analyzer measurements were repeated three times and averaged to obtain a high quality standard by which to judge the performance loss due to the low cost calibration circuit.

The next phase of the testing was to repeat the test with the addition of the experimental auto-calibration circuit in order to compare the high quality gold-standard control measurements, obtained previously for each delay line standard, against the answer obtained for the same delay lines when the auto-calibration circuit was utilized to provide access to on-board calibration standards, for periodic calibration of the on-line sensor, as well as to provide access to the delay lines themselves that are stand-ins for the material under test.

**Figure 5.**
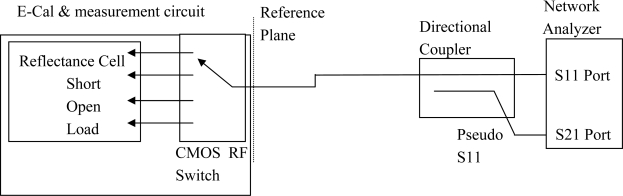
Shown is the testing configuration that was used to measure the performance of the proposed auto-calibration circuit.

To provide a representative method for testing the quality of the calibration circuit, “E-Cal”, that would be used by the field deployed sensing circuitry as well as to remove the bias that use of the low-cost sensor instrumentation would add, the network analyzer was configured as a stand-in replacement for the sensor instrumentation. To achieve this goal, the network analyzer was configured in a non-standard method by adding an external directional coupler that would redirect the reflected S11 signal, from reflectance cell, over to the S21 port thereby allowing the S21 port measurements, on the network analyzer, to provide a pseudo S11 measurements for each of the 4 selected measurements {open, short, load, delay-line}, as shown in Figure 5, with load being the delay or media filled coaxial test cell.

**Figure 6.**
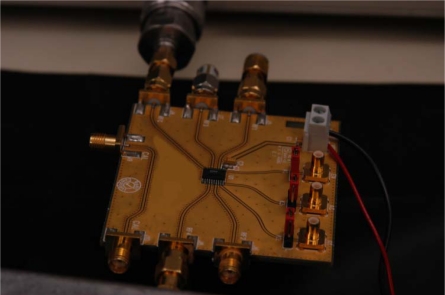
The experimental prototype of a low cost electronic calibration circuit. The circuit consists of insertion of a single-pole-4-throw 2.5 GHz bandwidth CMOS semiconductor switch which is inserted between the coaxial interconnecting cable and the sensing probe, per the schematic diagram as detailed in Figure 5.

The final requirement in the test configuration was to remove the effects of the added external directional-coupler from the network analyzer measurement, which was achieved by running a calibration on the network analyzer for a S21 response through-calibration with the reference plane located immediately before the E-Cal measurement circuit at the end of the interconnecting coaxial cable, per Figure 5. Thus, with this setup, the network analyzer S21 port measurements provided a high quality S11 measurement of the reflected power, from the delay line, in lieu of the reflectance cell, or one from one of the selected E-Cal’s internal calibration loads. This testing protocol ensured the tested accuracy of the system was limited to the errors created by the insertion of the E-Cal auto-calibration circuit before the reflectance cell and thereby provided a high quality measurement of the limitations of the proposed auto-calibration system based around the low-cost rf-switch. Of particular note was that in order to ensure a symmetric response between the various calibration loads as well as the reflectance cell, and thereby preserve the reference plane position, the circuit board layout should provide near equal path lengths between each of the switched ports, as shown in Figure 6.

## 5. Results and Conclusions

In the current configuration of the moisture sensors for use with cotton bales, the system takes an air-reference reading just after the bale has cleared the sensing antennas. This air reference is a necessary and critical part of the sensing design that is utilized to center the circuit due to changes in the internal signal delays and attenuations caused by temperature changes to the various system components and circuit elements. This air-reference technique has been very successful in commercial field trials, however, for many optimal locations in cotton gins for sensing of seedcotton moisture, the material flow does not stop at any time during processing, which thereby precludes the use of the necessary air-reference step. Thus, without some expensive mechanical isolation system, there is no way to obtain a material free calibration reading that could be used to remove the temperature drift and bias from the measurements. A similar problem occurs for reflectance measurements for frequency domain soil moisture sensors and other continuous on-line process control sensors. To alleviate this problem, this research examined the accuracy that could be obtained through the use of a low-cost off-the shelf SP4T, 4:1, microwave wide bandwidth rf-switch that could be used to periodically obtain access to several on-board calibration standards.

**Figure 7.**
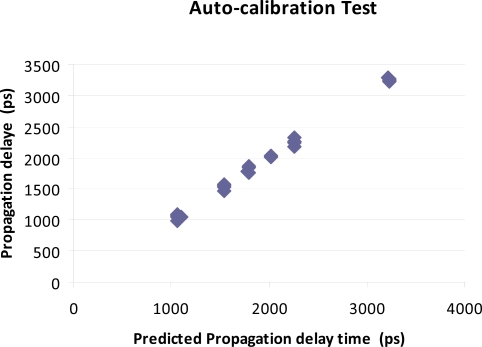
Results showing the correlation between in-circuit auto-calibration using on-board calibration standards switched in via microwave solid-state switch as compared to a traditional vector network analyzer calibration via traditional one-port S11 error correction method; testing utilized 4 replicated measures at 6 levels of delay.

The testing of the proposed low-cost rf-switch to form the heart of an electronic calibration system, E-Cal, revealed that due to cross-talk and lower between channel isolation, in comparison to a standard network analyzer, the best calibration that was obtainable was found to be the more limited use of a single response type calibration standard, utilizing only the open circuit load. The tests results, as detailed in Figure 7, do however suggest that the use of a single microwave switch, without a more elaborate configuration utilizing expensive directional couplers, does provide a reasonable low-cost alternative to ensure the accuracy of frequency domain based sensing systems that require an internal calibration, due to the nature of the installation. The proposed E-Cal technique provides a simple hardware implementation for electronic internal calibration when used in conjunction with microwave reflective sensing probes that support an extended bandwidth from 500 MHz through 2.5 GHz. Comparison testing utilizing the new proposed E-Cal technique was validated experimentally against known precision propagation delay standards via high accuracy network analyzer measurements. Results of the measured propagation delay with this type of automatic electronic calibration method was found to agree with the results provided through the use of a vector network analyzer when calibrated with a traditional S11 single port error correction calibration protocol. The accuracy of the proposed auto-calibration circuit was found to allow the system to provide measurements that were within 4% of the high quality network analyzer measurement with a 95% confidence, and a standard error of +/− 18.6 ps for the delay measurements. These results are shown in Figure 7.

In summary, this study found the following:

- A simple microwave 2:1 switch with a single open circuit calibration standard shows promise to be able to provide an on-board internal calibration to the microwave moisture sensor.
- The degradation from the high accuracy control measurements was less than 4% of the reading which would translate to less than 0.25% moisture content across the normal range of seedcotton moisture;
- The agreement between the test measurements and high accuracy control measurements provided a standard error of less than +/− 18 ps;
- The proposed microwave high-bandwidth CMOS rf switch provides a low-cost solution for incorporating automatic calibration into 100% on-line sensors that is suitable for cost-sensitive embedded process control sensors.

The results of this study suggests that the seedcotton moisture sensors can be effectively calibrated in the field through the use of a low-cost microwave rf switch, that is directly connected between the reflectance circuit, associated internal calibration loads, and the sensing circuitry. The techniques outlined in this study provide a low-cost solution to the problem of in-circuit calibration that can be utilized for a number of different frequency domain reflectance sensing applications where a calibration is not possible due to the nature of the installation. Such applications that will benefit from in-circuit calibration are moisture sensing of seedcotton in cotton gin processing and buried reflectance probes used in soil moisture sensing.
